# Effectiveness of computer-assisted cognitive training on cognitive function and activities of daily living in patients with post-stroke cognitive impairment

**DOI:** 10.3389/fnagi.2025.1590783

**Published:** 2025-08-20

**Authors:** Xiaoyang Feng, Xiaolin Sun, Jia Liu, Yan Li, Yunhai Yao, Jianming Fu, Xudong Gu

**Affiliations:** ^1^Department of Neurology, Zhejiang Province People’s Hospital Haining Hospital, Haining, China; ^2^Department of Rehabilitation Medicine, The Second Affiliated Hospital of Jiaxing University, The Second Hospital of Jiaxing, Jiaxing, China

**Keywords:** computer-assisted cognitive training, stroke, cognitive impairment, attention, execution, memory

## Abstract

**Objective:**

This study aimed to investigate the effects of computer-assisted cognitive training (CACT) on cognitive function and activities of daily living in patients with post-stroke cognitive impairment. Additionally, it aimed to explore the changes in specific cognitive domains before and after treatment.

**Design:**

The study was a double-blind, randomized, controlled trial.

**Setting:**

It took place in rehabilitation wards or outpatient clinics.

**Participants:**

Sixty patients with post-stroke cognitive impairment took part in the study.

**Interventions:**

Participants were randomly assigned to either the control (*n* = 30) or the intervention group (*n* = 30). Both groups received conventional rehabilitation and cognitive training, and the intervention group additionally received CACT.

**Main outcome measures:**

The primary outcome measures included the Mini-Mental State Examination (MMSE) and event-related potential (ERP) P300 for cognitive function, as well as the modified Barthel Index (MBI) for activities of daily living. Secondary outcomes were the Trail Making Test (TMT), the Symbol-Digit Modalities Test (SDMT), the Auditory Verb Learning Test-Huashan version (AVLT-H), the Boston Naming Test (BNT), and the Clock Drawing Test (CDT), which assessed cognitive function across specific domains.

**Results:**

Both groups showed significant improvements in MMSE, MBI, amplitude of P300, and latency of P300, with the intervention group demonstrating more pronounced improvements compared to the control group. In terms of specific cognitive domains, the intervention group exhibited greater improvements than the control group in TMT-A, TMT-B, and AVLT-H. Both groups showed improvements in SDMT and BNT, but the differences between the groups were not statistically significant. Additionally, there was no significant improvement in the CDT for either group before and after treatment.

**Conclusion:**

CACT was found to improve patients’ cognitive function, especially in areas of attention, executive function, and memory. It also effectively improved activities of daily living.

## Introduction

Stroke is a common condition among the elderly and is associated with high rates of morbidity, disability, and mortality. With the aging global population and the availability of data from the Global Burden of Disease Study 2019 (GBD 2019 Stroke Collaborators, 2021), stroke has emerged as a major public health concern. Cognitive impairment is one of the main symptoms of stroke. Although often overlooked in the early stages, it can significantly hinder the recovery of motor, speech, and other functions as the disease progresses. Ultimately, this can greatly reduce the patient’s quality of life (Godefroy et al., 2024; Kapoor et al., 2019; Blomgren et al., 2019; Gallucci et al., 2024; Stolwyk et al., 2024; Mole and Demeyere, 2020; Oksala et al., 2009).

A synthesis of studies from several countries shows that the prevalence of post-stroke cognitive impairment (PSCI) ranges from approximately 20 to 80% (Sun et al., 2014; Dong et al., 2021; He et al., 2023; Kaddumukasa et al., 2023; Weterings et al., 2023; Moliis et al., 2021), and the prevalence of post-stroke dementia is 38% (Sexton et al., 2019). However, these figures vary according to different countries, regions, ethnicities, and diagnostic criteria (Huang et al., 2022). Stroke is a brain injury, and acute cognitive dysfunction is common in the initial phase. Studies have shown that the prevalence of acute cognitive impairment after stroke is 49.6%, but the prevalence of cognitive impairment decreases to 34.2% when patients’ condition is stabilized at 3–6 months after stroke (Dong et al., 2021). Thereafter, cognitive impairment will likely persist for a long period of time. A study conducted in Sydney reported that the prevalence of mild cognitive impairment and dementia 3–6 months after stroke was 36.7 and 21.3%, respectively (Sachdev et al., 2006). Therefore, early identification and early intervention of PSCI are important to improve the overall prognosis of stroke patients and reduce the burden on families and society (Quinn et al., 2021; Cova et al., 2022).

Current pharmacological treatments for PSCI primarily include cholinesterase inhibitors and non-competitive N-methyl-D-aspartate receptor antagonists, but their efficacy is very limited. Furthermore, the development of new drugs presents significant challenges. As a result, attention has shifted toward non-pharmacological interventions, particularly cognitive rehabilitation training, which has been shown to significantly improve cognitive function in post-stroke patients (Zheng et al., 2020; Wang et al., 2021). However, traditional cognitive training is often monotonous, boring, and dependent on trained personnel. Many patients do not receive long-term treatment after discharge, resulting in diminished efficacy over time. With the popularization of computers, efforts have been made to integrate computer technology into cognitive rehabilitation. Clinically, computer-assisted cognitive training (CACT) not only gets rid of the limitations of treatment staff and venues but also improves patients’ compliance due to its fun and challenge. Therefore, it has received favorable feedback from both doctors and patients.

Previous studies have demonstrated the effectiveness of CACT in improving cognitive function in patients with Parkinson’s disease (Bernini et al., 2019), multiple sclerosis (Moustafaa et al., 2022), stroke (Yeh et al., 2022), mild cognitive impairment (Li et al., 2019), and subjective memory complaints (Pereira-Morales et al., 2018). However, few studies have investigated the optimal timing and efficacy of CACT in patients with cognitive impairment after stroke. To fill this research gap, this study enrolled patients with PSCI of 3–6 months duration and assessed patients’ global cognition, specific cognitive domains, and daily living activities before and after treatment to analyze the therapeutic effect of CACT. This study provides data to support the clinical use of CACT.

## Methods

### Study design

The study was a double-blind, randomized, controlled trial conducted in the rehabilitation department of a general hospital. The study was approved by a local hospital ethics committee and adhered to the principles of the Declaration of Helsinki. All participants were informed and signed an informed consent form before enrollment.

### Participants

The inclusion criteria for participants in the study were as follows: (a) meeting the stroke diagnostic criteria of Diagnostic Points of Various Major Cerebrovascular Diseases in China 2019 and confirmed by cranial CT or MRI; (b) meet the diagnostic criteria of PSCI without dementia, as defined in the Expert Consensus on the Management of Cognitive Impairment after Stroke 2021; (c) first stroke with a unilateral lesion, hemiparesis on one side, National Institutes of Health Stroke Scale (NIHSS) score ≤ 7, and a disease duration of 3–6 months; (d) no fever and in stable condition; (e) age greater than 18 years; and (f) an elementary school education or above, with the ability to understand and cooperate with the rehabilitation training and assessments conducted in this study.

The exclusion criteria were (a) coma, tracheotomy, unstable vital signs, or serious concomitant medical conditions (e.g., heart, lung, liver, and kidney diseases); (b) factors affecting rehabilitation training or assessment, such as visual or hearing impairment, aphasia, recent fracture, severe cognitive dysfunction, or psychiatric symptoms; (c) other conditions affecting cognitive function, such as encephalitis, traumatic brain injury, depression, and Parkinson’s disease; (d) taking drugs that affect cognitive function, such as benzodiazepines; (e) having a history of cognitive disorders or serious psychiatric illness; (f) currently participating in other research studies; and (g) refusing to sign the informed consent form or cooperate with assessment and treatment.

A total of 60 cases of stroke patients who met the criteria and were admitted to the outpatient or inpatient departments of the Rehabilitation Medicine Centre from April 2023 to October 2024 were selected. Participants were randomly assigned to either the control group or the intervention group, with 30 cases in each group.

### Interventions

Patients in the two groups received conventional medication and rehabilitation therapy. The medication regimen included treatments for blood pressure control, blood sugar control, lipid regulation, blood circulation improvement, and nerve nutrition. Rehabilitation therapy included good posture, the Bobath technique, motor relearning, occupational therapy, daily living skills training, and traditional cognitive rehabilitation training. The rehabilitation sessions were conducted 2 h per day for 6 days a week for 4 weeks.

In the intervention group, all patients also received CACT treatment for 20 min a day for 6 days a week for 4 consecutive weeks. CACT adopts the equipment of the Flex Table digital OT assessment and training system and selects the appropriate cognitive training items and difficulty according to the results of the cognitive function assessment. The training includes the following programs, among others: (1) Attention Coordination Training: This program trains the patient’s attention. Cakes will fall from different directions onto the computer screen, and the patient will be asked to select the correct plate with which to catch them. (2) Virtual Kitchen: This program trains the patient’s executive function. Patients need to complete the entire cooking process on the computer, including holding, cleaning, dicing, and cooking tomatoes. (3) Memory Matrix Training: This program trains the patient’s memory. Nine or more squares appear on the computer screen, some of which light up briefly. The patient must remember the location of the lit squares and select them accurately afterward. (4) Arithmetic reasoning: This program improves the patient’s ability to perform calculations. Two sets of numbers or equations are displayed side by side on the screen. The patient is asked to judge the relationship between the values on the left and right, answering by clicking “>“, “<“, or “=“. (5) Jigsaw Rapid Matching Training: This program trains the patient’s visuospatial ability. First, the system shows a complete picture, which it then divides into several puzzle pieces. The puzzle pieces must then be moved and reassembled into the original picture. (6) Categorical Cognitive Training: This program trains the patient’s language ability. Several groups of pictures are presented to the left of the screen alongside a word on the right. The patient is asked to read the word aloud and identify the matching target picture from the pictures on the left. The therapist assisted patients as needed throughout the process, ensuring patients could complete tasks successfully. The difficulty of the training gradually increased as the patient’s cognitive function improved.

### Outcome measurements

Efficacy was assessed by a rehabilitation specialist who was blinded to the subgroups before and after 4 weeks of treatment.

#### Primary outcome measures

Cognitive function: The Mini-Mental State Examination (MMSE) was used to assess the general cognitive level of the patients. The version chosen was the Chinese version of the MMSE revised by Zhang Mingyuan, which takes 5–10 min and covers temporal and spatial orientation, immediate and delayed recall, attention and calculation, and verbal ability. The MMSE has a maximum score of 30. The higher the score, the better the cognitive function. The event-related potential (P300) is a bioelectrical response to central nervous system sensory stimulation that reflects the patient’s cognitive function (Liang et al., 2025). Measurements are amplitude and latency. The higher the amplitude and the shorter the latency, the better the cognitive function.

Activities of daily living (ADLs): The modified Barthel Index (MBI) was used to assess patients’ ability to perform ADLs, which consisted of 10 items, including eating, bathing, grooming, dressing, bowel control, toileting, transferring, walking on level surfaces, and walking up and down stairs. Each item was rated on a 5-point scale with a maximum total score of 100. The higher the score, the greater the level of independence in ADLs.

#### Secondary outcome measures

Attention and executive functions: The Trail Making Test (TMT) and the Symbol-Digit Modalities Test (SDMT) were used to assess attention and executive functions. The TMT is divided into two parts, A and B. The time taken to complete each task is recorded, with shorter completion times indicating better attention and executive function. For the SDMT, participants are required to fill in the corresponding symbols underneath the series of numbers as quickly as possible within 90 s. The more correctly the symbols are filled in, the faster the information can be processed.

Memory: The Auditory Verb Learning Test-Huashan version (AVLT-H) was used to assess memory function. The greater the number of words, the better the memory function.

Language: The Boston Naming Test (BNT) was used to assess language function. The greater the number of correct answers, the better the language function.

Visuospatial ability: The Clock Drawing Test (CDT) (Rouleau et al., 1992; Yamauchi et al., 2024) was used to assess visuospatial function. The higher the score, the better the visuospatial function.

### Statistical analysis

Data were analyzed using Statistical Packages for Social Sciences (SPSS) version 25.0. Categorical variables were presented as frequency counts (percentages) and compared using the Chi-squared test (*x^2^*). Continuous variables were expressed as mean ± standard deviation for normally distributed data or median (interquartile range) for non-normally distributed data. Prior to comparison, the Shapiro–Wilk test was used to verify the normality of the distribution. The t-test was applied to compare numerical variables that follow a normal distribution. Paired samples t-test was used to compare the values before and after treatment in each group. An independent sample t-test was used to compare the values between the control and intervention groups. The Wilcoxon signed-rank test was used to compare numerical variables that did not follow a normal distribution. Statistical significance was defined as a *p*-value of < 0.05.

## Results

A total of 60 patients completed the study. The demographic and baseline characteristics of the two groups of patients are summarized in Table 1. There were no significant differences in the baseline characteristics between the control and intervention groups, suggesting that the groups were comparable at the start of the study. Throughout the study, no serious adverse effects were reported. Some patients reported mild shoulder pain that resolved with rest.

**Table 1:** Demographics and baseline characteristics.

Variables	Control group (*N* = 30)	Intervention group (*N* = 30)	*x^2^/t/Z*	*p-*value
Age(years), median (IQR)	64.5(15.0)	63.5(14.0)	−0.311	0.756
Sex				
Female, N(%)	18(60.0%)	20(66.7%)	0.287	0.592
Male, N(%)	12(40.0%)	10(33.3%)		
Education level				
Primary school, N(%)	3(10.0%)	1(3.3%)	0.268	0.605
Junior middle school and above, N(%)	27(90.0%)	29(96.7%)		
Disease duration (day), mean ± SD	126.3 ± 24.2	132.7 ± 26.5	−0.988	0.327
Stroke type				
Ischemic stroke, N(%)	20(66.7%)	14(46.7%)	2.443	0.118
Hemorrhagic stroke, N(%)	10(33.3%)	16(53.3%)		
Side of paresis				
Right, N(%)	16(53.3%)	13(43.3%)	0.601	0.438
Left, N(%)	14(46.7%)	17(56.7%)		

Data are shown as mean ± SD or median (IQR) or N (%). SD, standard deviation; IQR, interquartile range; N, number.

### Primary outcome

For MMSE, both the control group (*p* < 0.01) and the intervention group (*p* < 0.01) showed significant improvement after treatment compared to before treatment. The intervention group demonstrated better results compared to the control group (*p* < 0.01) (Table 2).

**Table 2:** Comparison of cognitive functioning and activities of daily living before and after treatment.

Variables	Time	Total (*N* = 60)	Control group (*N* = 30)	Intervention group (*N* = 30)	*t*/*Z*	*p-*value
MMSE (score), median (IQR)	Pre	24.0(2.0)	24.0(2.0)	24.0(2.0)	−1.479	0.139
	Post	27.0(2.0)	26.0(1.0)^*^	28.0(1.0)^*a^	−4.106	0.000
TMT-A (s), mean ± SD	Pre	113.5 ± 17.9	114.3 ± 18.6	112.6 ± 17.4	0.351	0.727
	Post	102.2 ± 12.5	105.8 ± 12.5	98.7 ± 11.7^*a^	2.273	0.027
TMT-B (s), mean ± SD	Pre	249.0 ± 45.8	250.7 ± 42.8	247.2 ± 49.3	0.288	0.774
	Post	206.1 ± 32.7	218.5 ± 31.5^*^	193.6 ± 29.4^*a^	3.166	0.002
SDMT (score), median (IQR)	Pre	22.0(1.0)	22.0(1.3)	22.0(0.5)	−1.048	0.295
	Post	23.0(1.0)	23.0(1.0)^*^	23.0(2.3)*	−1.133	0.257
AVLT-H (score), median (IQR)	Pre	3.0(0.0)	3.0(0.0)	3.0(1.0)	−0.106	0.915
	Post	3.0(1.0)	3.0(0.0)	3.0(1.0)^*a^	−3.018	0.003
BNT (score), mean ± SD	Pre	20.9 ± 3.4	20.7 ± 3.5	21.1 ± 3.3	−0.380	0.705
	Post	22.9 ± 3.1	22.6 ± 2.8^*^	23.2 ± 3.4^*^	−0.751	0.456
CDT (score), median (IQR)	Pre	7.5(5.0)	8.0(5.0)	7.0(5.0)	−0.172	0.863
	Post	8.0(4.0)	8.0(5.0)	7.5(3.3)	−0.091	0.928
MBI (score), median (IQR)	Pre	38.0(2.8)	38.0(6.3)	37.0(1.0)	−1.558	0.119
	Post	58.0(6.0)	53.5(2.0)^*^	59.0(1.0)^*a^	−4.292	0.000
Amplitude of P300 baseline (mv), mean ± SD	Pre	5.2 ± 1	5.4 ± 1.1	5.1 ± 0.9	0.762	0.449
	Post	7.4 ± 1.7	6.9 ± 1.5^*^	7.9 ± 1.6^*a^	−2.661	0.010
Latency of P300 baseline score (ms), mean ± SD	Pre	380.7 ± 26.1	378.7 ± 25.2	382.6 ± 27.3	−0.585	0.561
	Post	353.7 ± 22.7	359.9 ± 21.6^*^	347.4 ± 22.4^*a^	2.197	0.032

^∗^*p* < 0.05 vs. before treatment; ^a^ *p* < 0.05 vs. control group.

N, number; IQR, interquartile range; SD, standard deviation; MMSE, Mini-Mental State Examination; TMT, Trail Making Test; SDMT, Symbol-Digit Modalities Test; AVLT-H, Auditory Verb Learning Test-Huashan version; BNT, Boston Naming Test; CDT, Clock Drawing Test; MBI, modified Barthel Index; Pre, preintervention; Post, postintervention.

For MBI, both the control group (*p* < 0.01) and the intervention group (*p* < 0.01) showed significant improvement after treatment compared to before treatment. The intervention group demonstrated better results compared to the control group (*p* < 0.01) (Table 2).

For the amplitude of P300, both the control group (*p* < 0.01) and the intervention group (*p* < 0.01) showed significant improvement after treatment compared to before treatment. The intervention group demonstrated better results compared to the control group (*p* = 0.010).

For the latency of P300, both the control group (*p* < 0.01) and the intervention group (*p* < 0.01) showed significant improvement after treatment compared to before treatment. The intervention group demonstrated better results compared to the control group (*p* = 0.032) (Table 2).

### Secondary outcome

For TMT-A, the control group showed no significant improvement after treatment (*p* = 0.089). In contrast, the intervention group showed significant improvement after treatment (*p* = 0.002).

For TMT-B, both the control group (*p* = 0.001) and the intervention group (*p* < 0.01) showed significant improvement after treatment compared to before treatment. The intervention group demonstrated better results compared to the control group (*p* = 0.002) (Table 2).

For SDMT, both the control group (*p* = 0.032) and the intervention group (*p* = 0.001) showed significant improvement after treatment compared to before treatment. However, there was no significant difference between the two groups (*p* = 0.257) (Table 2).

For AVLT-H, the control group showed no significant improvement after treatment (*p* = 0.439), while the intervention group showed significant improvement (*p* = 0.001) (Table 2).

For BNT, both the control group (*p* = 0.029) and the intervention group (*p* = 0.008) showed significant improvement after treatment compared to before treatment. However, there was no significant difference between the two groups (*p* = 0.456) (Table 2).

For CDT, there was no significant improvement in either the control group (*p* = 0.530) or intervention group (*p* = 0.419) (Table 2).

## Discussion

Our study explored the effects of CACT on patients with PSCI, aiming to provide valuable insights into the potential benefits of this treatment. The results revealed the following: (1) both the control group and the intervention group exhibited significant improvements in cognitive function and ADLs after treatment. Notably, CACT provides additional benefits over standard rehabilitation methods. (2) As the P300 is a neurophysiological measure of cognitive processing, our study further supports the finding that CACT enhances cognitive function. (3) In further exploration of specific cognitive domains, we found that, relative to conventional rehabilitation therapy, CACT combined therapy has more significant advantages in improving patients’ attention, executive function, and memory. However, its impact on visuospatial abilities, language, and processing speed may be less pronounced.

Cognitive impairment is one of the major functional deficits in stroke patients, which significantly affects prognosis and quality of life (Einstad et al., 2021). After years of clinical practice, it has been found that the efficacy of treatment for moderate-to-severe cognitive impairment is poor. Clinicians should focus on early detection and intervention of mild cognitive impairment, thereby slowing down the further progression of cognitive impairment and reducing the prevalence of dementia (Jia et al., 2021; Anderson, 2019; Chau et al., 2023). For the treatment of cognitive function, medication is the foundation, while rehabilitation is the focus. Traditional rehabilitation strategies such as physical therapy (Liu-Ambrose et al., 2022; Gjellesvik et al., 2021), occupational therapy (Gibson et al., 2022; García-Pérez et al., 2024), and cognitive training (Kim and Jang, 2021) have shown some success but often require intensive resources and can be limited by the availability of trained personnel. CACT is a novel computerized cognitive training method that is delivered via an automated and engaging platform. Some studies have found that the effects of CACT have a more pronounced advantage in newer cognitive training modalities, such as virtual reality (Xiao et al., 2022).

Chen et al. (2024) found that both CACT and conventional cognitive training improved patients’ global cognitive functioning and activities of daily living, and the efficacy of both was comparable. It suggests that CACT has the potential to replace conventional cognitive training. Similarly, Ho et al. (2022) applied CACT to stroke survivors with a treatment frequency of 20 min per session, twice a week for 12 weeks. Outcome measures included the Mini-Mental State Examination (MMSE), Montreal Cognitive Assessment (MOCA), backward digit span test, and SDMT. The results showed that CACT significantly improved global cognitive function and specific cognitive domains (information processing speed and working memory). The effect on activities and participation was not observed. We believe that the frequency of treatment in the study was low and that focused treatment may have been more efficacious. Although there was also a significant improvement in all three MoCA subtests, namely, attention, naming, and delayed recall, in the study, we recommend a more specific assessment to validate the effectiveness of the treatment. Kazinczi et al. (2024) reviewed several studies and concluded that CACT significantly improved patients’ scores on the Digit Span Backward Test and Visual Span Forward Test. It is suggested that CACT has the effect of promoting the recovery of working memory. This result is consistent with our study. Svaerke et al. (2019) showed that CACT could be beneficial in patients with visuospatial neglect or homonymous hemianopia in the subacute phase after stroke. However, our study, using the BNT to assess visuospatial ability, did not observe a positive effect. The reason may be that the patients included in this study had a lesser degree of impaired visuospatial ability or that the treatment was relatively short-term.

Previous studies have shown that cognitive deficits in stroke patients mainly involve attention, memory, verbal function, and visuospatial abilities (Moliis et al., 2021; Shendyapina et al., 2022; Hua et al., 2023; Siow et al., 2024; Lugtmeijer et al., 2021). Additionally, impairments in executive function and visuospatial hypoplasia are associated with patient survival (Oksala et al., 2009). However, we should recognize that these assessments have some bias. Tools used in the study, such as the MMSE and MOCA, require a certain level of cooperation from participants, which may exclude patients with severe cognitive impairment, communication disorders, visual impairment, or neglect syndrome. This has also led to low detection rates and a lack of truly valid assessments of impairments in specific cognitive domains such as verbal functioning.

CACT’s effectiveness in improving cognitive function can be attributed to several mechanisms. First, CACT can attract patients to accept and use it because of its convenience and fun, thus improving treatment adherence. Second, it can promote neural function remodeling through rich multisensory repetitive training, thus improving cognitive function (De Luca et al., 2018; O'Sullivan et al., 2022; Sihvonen et al., 2022). Functional MRI studies by Filippi et al. (2012) suggest that CACT can modulate the activity of cognitive brain regions, such as posterior cingulate cortex (PCC)/precuneus cortex and dorsolateral prefrontal cortex (PFC), to enhance the recruitment of brain networks, thereby improving patients’ attention, information processing, and executive function. Recent studies have emphasized the importance of brain connectivity, rather than isolated brain regions (Godefroy et al., 2024; Thiebaut de Schotten et al., 2020; Eriksson et al., 2023). Lisanne et al. suggested that CACT could increase executive function by decreasing the connection between the default mode network (DMN) and the frontoparietal network (FPN) (Ten Brinke et al., 2021). Finally, CACT records and analyzes the patient’s errors in the test in real time, guiding us to adjust the training module and difficulty. This personalized approach may further contribute to its effectiveness.

### Study limitations

This study has several limitations. First, this is a single-center study with a limited representative population, and the results are not representative of populations in other regions and of different races. Second, there was no long-term follow-up in the study, and it was not possible to estimate the duration of the efficacy of CACT on different cognitive domains and whether repeated training is needed over a long period of time. Finally, the study lacked a placebo control group, and non-cognitive computer-assisted training could be introduced in future studies to further confirm the effectiveness of CACT.

## Conclusion and prospects

Our research findings suggest that CACT can improve patients’ cognitive function, particularly in terms of attention, executive function, and memory. It can also effectively enhance activities of daily living. The advantages of CACT are as follows: it is engaging and enjoyable; it allows patients to enhance their cognitive abilities through play; it can record and analyze cognitive performance in real time, enabling users to adjust the game’s difficulty according to their progress; and it reduces dependence on therapists to some extent. However, CACT also has certain limitations: it requires equipment and a certain degree of therapist guidance; it is limited to patients with mild-to-moderate cognitive impairment who can understand the rules; users need a certain degree of physical function and strength; and it lacks the emotional support of interpersonal communication. Our future research direction is to develop CACT for remote home treatment and monitor its effectiveness. Meanwhile, the assessment of executive function aspects such as task switching ability and inhibitory control will be further refined.

## Data Availability

The original contributions presented in the study are included in the article/supplementary material, further inquiries can be directed to the corresponding author.
